# Physiological and Pathophysiological Aspects of Primary Cilia—A Literature Review with View on Functional and Structural Relationships in Cartilage

**DOI:** 10.3390/ijms21144959

**Published:** 2020-07-14

**Authors:** Friedrich Barsch, Tanja Niedermair, Andreas Mamilos, Volker H. Schmitt, David Grevenstein, Maximilian Babel, Thomas Burgoyne, Amelia Shoemark, Christoph Brochhausen

**Affiliations:** 1 Institute of Pathology, University Regensburg, 93053 Regensburg, Germany and Institute of Exercise and Occupational Medicine, Department of Medicine, University of Freiburg, 79106 Freiburg, Germany; friedrich.barsch@uniklinik-freiburg.de; 2 Institute of Pathology, University Regensburg, 93053 Regensburg, Germany; Tanja.Niedermair@ukr.de (T.N.); Andreas.Mamilos@ukr.de (A.M.); Maximilian.babel@stud.uni-regensburg.de (M.B.); 3 Cardiology I, Centre for Cardiology, University Medical Centre, Johannes Gutenberg University of Mainz, 55122 Mainz, Germany; volker.schmitt@unimedizin-mainz.de; 4 Department for Orthopedic and Trauma Surgery, University of Cologne, 50923 Köln, Germany; david.grevenstein@uk-koeln.de; 5 Royal Brompton Hospital and Harefield NHS Trust, SW3 6NP London and UCL Institute of Ophthalmology, University College London, London EC1V 9EL, UK; t.burgoyne@ucl.ac.uk; 6 Royal Brompton Hospital and Harefield NHS Trust, University of Dundee, Dundee DD1 4HN, UK; a.shoemark@dundee.ac.uk

**Keywords:** Primary cilia, cartilage, osteoarthritis, mechanotransduction

## Abstract

Cilia are cellular organelles that project from the cell. They occur in nearly all non-hematopoietic tissues and have different functions in different tissues. In mesenchymal tissues primary cilia play a crucial role in the adequate morphogenesis during embryological development. In mature articular cartilage, primary cilia fulfil chemo- and mechanosensitive functions to adapt the cellular mechanisms on extracellular changes and thus, maintain tissue homeostasis and morphometry. Ciliary abnormalities in osteoarthritic cartilage could represent pathophysiological relationships between ciliary dysfunction and tissue deformation. Nevertheless, the molecular and pathophysiological relationships of ‘Primary Cilia’ (PC) in the context of osteoarthritis is not yet fully understood. The present review focuses on the current knowledge about PC and provide a short but not exhaustive overview of their role in cartilage.

## 1. Introduction

**Figure 1:**
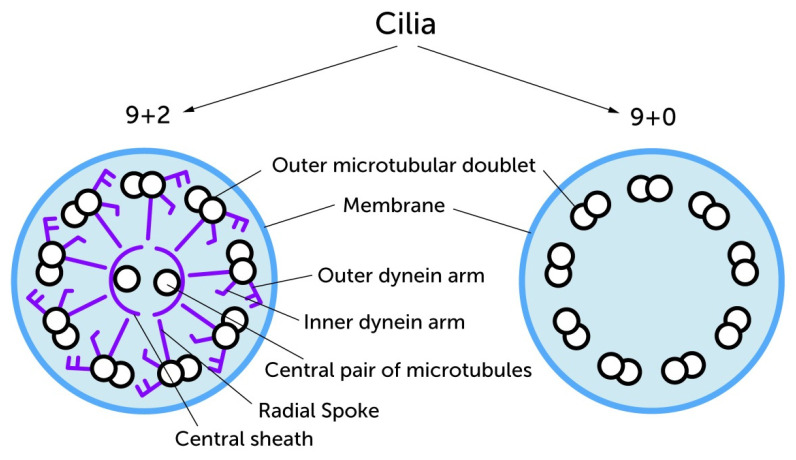
The early classification of cilia depended largely on differences in ultra structure. The 9 + 2 microtubule arrangement is most commonly found in motile cilia which carry additional structural proteins in their axonemes especially the radial spoke, the inner and the outer dynein arms (purple colored) to perform their beating function. These structural proteins are missing in most of the primary monocilia. Both groups have in common that the basic framework is formed by microtubule doublets. Image modified from Takeda and Narita (2012) [15].

Cilia were first described in the 17th century, when Antoni van Leeuwenhoek worked with a prototype microscope and detected moving, elongated structures on Protozoa, which he described as “little legs” [1]. It is well known that cilia occur in nearly all human tissues [2,3,4,5]. Early ultrastructural investigations revealed cilia have either a cross sectional 9 + 2 or 9 + 0 microtubular arrangement. Whereas the axoneme of 9 + 0 cilia contains 9 microtubular doublets cilia with the 9 + 2 arrangement contain additional structures. The ciliary components include 9 microtubular doublets with inner and outer dynein arms, a central pair of microtubules (the +2 component) surrounded by a central sheath and radial spokes radiating between the doublets and central pair [6,7]. This 9 + 2 versus 9 + 0 structural difference resulted in the initial classification of cilia as ‘Motile Cilia’ or ‘Primary Cilia’ (PC) (Figure 1). In this classification, PC are understood to occur as a single cilium per cell (monocilia), whereas there are typically multiple motile cilia per cell. However, the classification based on cross-sectional structures can be misleading. It has been shown that there are 9 + 0 monocilia that are motile (for example in the embryonic node) and 9 + 2 cilia that are non-motile (for example in the mammalian cochlea) [8]. Research in the last century has focused primarily on the functions of the multiple motile cilia, leading to poorer understanding of the specific functions of the PC. Historically, PC were considered as rudimentary cell organelles [6,9]. However, the functional importance of PC in different tissues has become increasingly clear over time [1]. Modern molecular analysis of ciliary proteins and genes have detected relationships between important molecular pathways (such as Wnt (Wingless/int) and Hh (Hedgehog) signaling) and the PC. The process of ciliogenesis, its connection with the cell cycle, Intraflagellar Transport (IFT) and endo- and exocytosis at the PC site, have been intensively explored. Today, PC are understood as multifunctional, solitary cell organelles, which coordinate signaling at the interface between the extra- and intracellular space. The growing knowledge about elementary processes, in which PC are involved provides evidence for associations between ciliary dysfunctions and the occurrence of several pathologies. These include chronic conditions such as osteoarthritis and rare inherited diseases caused by cilia dysfunction termed ‘Ciliopathies’ [10,11,12,13]. Ciliopathies are typically multi-organ syndromes with features such as cystic kidneys, situs inversus, retinal degeneration, sensoneural deafness and developmental delay. Genetic ciliopathies are often associated with skeletal anomalies, such as polydactyly, shortened limbs and thoracic deformities. These observations suggest that PC have a role in the development and homeostasis of mesenchymal tissues. PC have been shown to contribute to several processes that affect the morphogenesis of cartilage and bones during embryological development. Adequate cell proliferation, cell differentiation, (stem-)cell maintenance, cell migration and cell polarity are interconnected with the proper function of the PC. In mature articular cartilage PC recognize, coordinate and regulate a number of cellular signals and processes. For example, PC have the capacity to sense extracellular mechanical and molecular stimuli and relay it to the cell to promote a cellular response [14]. Thus, PC are important signaling mediators between the extracellular environment (including the extracellular matrix (ECM)) and the intracellular compartments.

This review provides an overview of the recent knowledge of physiological and pathophysiological aspects of PC with a special focus on chondrocyte PC. The work reviewed here is based on previously published literature and considers experimental studies as well as conjectures formed within other reviews. The literature search was based on PubMed and included the key words “primary cilia”, “cartilage”, “ciliogenesis”, “intraflagellar transport” and “osteoarthritis.”

## 2. Physiological Aspects of Primary Cilia—Form Follows Function

### 2.1. Structural and Functional Features of Primary Cilia

PC are solitary, hair-like cell organelles, that protrude from the cell surface with a high surface to volume ratio [5,6]. The PC is a multifunctional organelle containing an ensemble of well-organized structural elements, receptors and proteins, which interact in a well-orchestrated manner. The observation PC are located in proximity to other intracellular compartments such as the Golgi apparatus, indicates that PC are coupled and involved in protein transport mechanisms. The interactions between ciliary and extraciliary components are regulated by signaling pathways along the PC. Several pathways, which are responsible for multiple cellular and tissue developing processes are connected to PC. These include Shh-, Wnt-, Notch-, RTK-, TGF-β-, TRPV4-, mTOR-, PDGFR- and Hippo-pathways that are reviewed elsewhere [16]. The activation of pathways through intra- or extracellular chemical or mechanical factors triggers intracellular signal cascades such as an increase of Ca^2+^ or the induction of Gli transcription factors.

The ciliary surface carries special receptors and ion channels, which are responsible for several functional processes at the ciliary site [7,17]. In this context, G-protein coupled receptors (GPCR’s), PTCH1, ECM-receptors, integrins, connexin 43 and ion channels (PIEZO, TRPV4) have been detected on the ciliary membrane as well as TGF-β receptors I & II and Polycystin channels PC1 and PC2 [5,16,17,18,19]. The high surface to volume ratio supports greater receptor diversity which reflects the multifunctional role of PC [20]. The area between the cell membrane and the ciliary membrane forms a deeply invaginated fold—the so-called ‘ciliary pocket‘ (CP) [14,18,21,22]. The CP surrounds the base of the PC and includes Clathrin Coated Vesicles (CCV) and AP2 adaptor complex, implying that it is a site for endocytosis [6,14,16,22].

**Figure 2:**
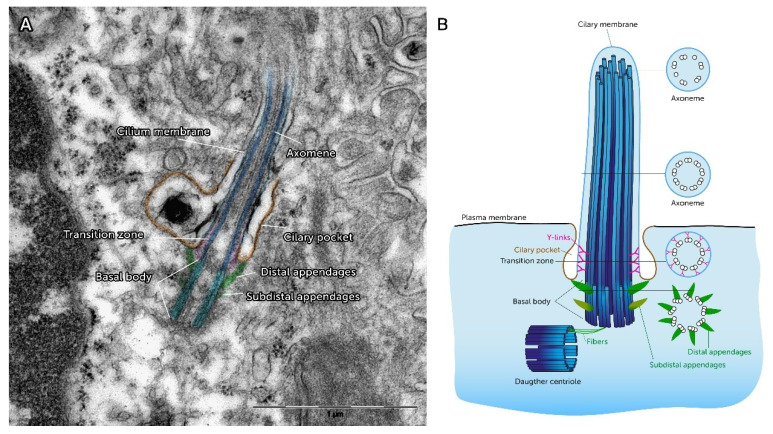
Structural features of Primary Cilia (**A**) Color-coded original electron microscopic image of a longitudinal section of a primary cilium show the typical structure of these cell organelles: basal body in bright blue, axoneme in dark blue, distal appendages in green, the transition zone in pink and the ciliary pocket in orange. (scale bar 1 µm). (**B**) The schematic image of the primary cilium gives information about the change of the cross-sectional structure in the course of the ciliary shaft. Recent three-dimensional reconstructions on epithelial primary cilia show that the structure in the tip of the cilium leaves the typical 9 + 0 pattern of the shaft. Instead some microtubule doublets separate into singular microtubules at the tip. Image modified from Ke et al. (2014) [31].

Longitudinal electron microscopical sections reveal that the PC is pervaded by microtubules (Figure 2). This inner framework of the PC known as the ‘Axoneme,’ elongates continuously from the ‘Basal Body’ (BB). The BB represents a barrel-like, cylindrical structure of ~0.5 µm in length and 0.2 µm in width which is localized beneath the cell membrane [23]. In cross section it is characterized by microtubular triplets in a ninefold symmetrical pattern which is thought to be a template for the assembly of the axoneme and its 9-fold symmetry (Figure 2) [24]. The area between the elongated ciliary shaft and the BB is characterized by Y-shaped protein structures that link the microtubule doublets to the ciliary membrane. As freeze-fracture electron microscopic imaging revealed, these connections are arranged around the circumference of the axoneme and define the proximal end of the ciliary membrane [25]. Because this area is arranged between the elongated PC and the BB, it is called ‘Transition Zone’ (TZ). A radial arrangement of Transition Fibers (TF) link the BB to the CP plasma membrane, can be detected in the TZ [6]. The molecular architecture of TF contains proteins like Cep164 and Ccdc123/Cep123 [25]. By the molecular structure of the TF, the TZ creates a physical and functional filter for proteins and vesicles [25,26]. Thereby, the TZ plays a role in the coordination and regulation of selective protein loading, membrane trafficking and endocytosis [26,27]. The BB shows a basal foot at its proximal site, which consists of subdistal appendages. The subdistal appendages contain Ninein as a characteristic structure protein [25]. Underneath the subdistal appendages the BB is connected with the daughter centriole via fibers [28]. The whole anchoring complex has significantly more features than just the mechanical fixation of the axoneme. Reiter et al. reviewed multiple proteins were targeted at the TF’s site, for example tubulin folding Cofactor C, which is necessary to build up the axoneme [25]. The complex structure and substructure of PC is a morphological correlate of their functional involvement in physiological and pathophysiological processes, such as the modulation of inflammatory reactions and the epithelial to mesenchymal transition type II [29,30]. Thus, it becomes clear a well-orchestrated interaction of the morphological components are crucial for the proper functions of the PC. As a consequence, structural changes of PC lead to several pathologies (see below).

### 2.2. Ciliogenesis—A Cell Cycle Dependent Process

**Figure 3:**
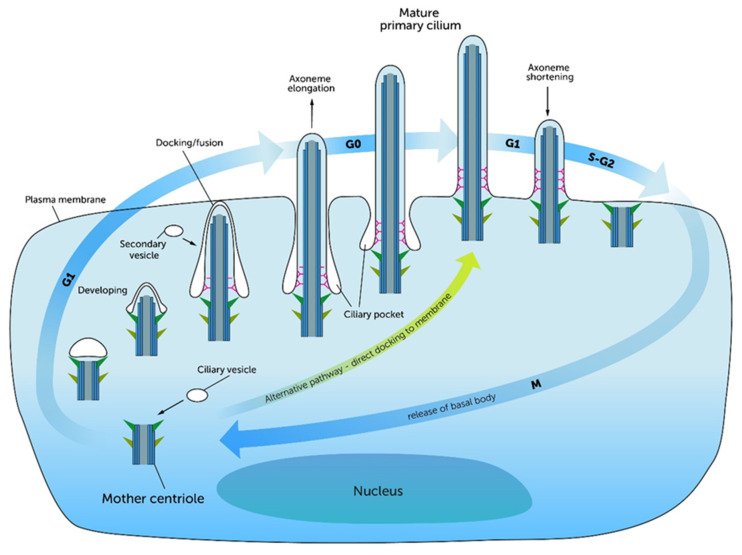
Starting from the mother centriole, ciliogenesis begins intracellularly. In the G1 phase of the cell cycle the mother centriole docks to ciliary vesicles or alternatively directly to the cell membrane. The resulting Basal Body forms the basis of the elongating cilium. By nourishing the ciliary membrane with secondary vesicles, the formation of the transition zone takes place. After fusion with the cell membrane, the ciliary gate is formed and the extension of the axoneme starts. The cilium reaches its full length and complete structure in the G0 phase of the cell cycle. The complete ciliary length is achieved through an equilibrium of assembling and disassembling processes. When the cell re-enters the cell cycle, the axoneme is degraded and the Basal Body is formed back to the mother centriole. Thus, the centriole is ready to run through mitosis again. Image modified from Molla-Herman et al. (2010) [22].

The biogenesis of cilia is commonly known as ‘Ciliogenesis.’ Ciliogenesis is defined as a multistep process that starts within the cell and ends with the complete formation of the functional cilium (Figure 3) [25,32]. Ciliogenesis is restricted to and reciprocally linked to the G1/G0 phase of the cell cycle [27,32,33,34,35]. The division of the mother and daughter centrioles occurs after mitosis. Centriolar satellites spread out of the achromatic mother centriole, join the microtubule triplets and form the BB. After the formation of the BB, the early stages of ciliogenesis can proceed via an ‘intracellular pathway’ or an ‘extracellular pathway.’ The intracellular pathway is characterized by the docking of ciliary vesicles at the distal end of the centriole deep in the cytoplasm [20,24,25,27,32]. In this case, the ciliary vesicles are derived from the Golgi apparatus. These steps are mostly described by morphological observations. Sorokin et al. and Tucker et al. described the ciliary bud which appears at the BB, from which the ciliary shaft is elongated [36]. They demonstrated that the BB connects itself with its distal end to a pellicle forms a cuff and in turn builds an epiplasmic cap, which fuses with the BB [37]. Simultaneously to the formation of the BB and the invagination of ciliary vesicles, the BB migrates to the cell surface and fuses with the cell membrane [25,27,33].

In contrast to the intracellular pathway, the extracellular pathway is characterized by direct docking of the centriole to the cell’s membrane without intervening intracellular stages [32,38]. One explanation for the occurrence of these two different pathways is that the cell membrane stays adhered to the mother centriole during mitosis. Therefore, the centriole can directly start ciliation in this daughter cell, while the centriole of the other daughter cell needs to be complemented with vesicles from the Golgi apparatus to become a BB [38]. However, the docking of the BB at the cell membrane is associated with actin filaments and components of the TZ, indicating that the TF and the TZ form the ciliary gate during these steps [25,27]. In this process, correct docking and the apical-basal arrangement are promoted by a protein complex called Par3-Par6-aPKC [33]. Afterwards, the formation of the PC takes place by the elongation of the axoneme [20,25,32]. Here, the extension starts from the tip of the cilium by a mechanism known as ‘Intraflagellar transport’ (IFT). IFT, described in detail in the subsequent section of this manuscript, plays a key role in ciliary assembly and disassembly. Even before it comes to the elongation of the cilium, proteins, which are necessary for IFT, accumulate around the mother centriole [24]. In the G1/G0 phase the PC grows to a defined length, which is then maintained through an equilibrium of assembling and disassembling processes [32]. Interestingly, the resorption of the PC is initiated at the re-entry into the cell cycle where the molecular balance tips to disassembling processes, which includes smaller IFT trains carrying less protein to the tips [27,32,33]. Ciliogenesis in growing mammalian cells is consistent with cell cycle phases [33,39,40]. While this process occurs during G0 and late G1 phase, the deciliation begins, when the cell re-enters the cell cycle. This is mediated by molecular signals, which influence the ciliary pathways [33,39,40]. These observations were one decisive factor for recent studies to focus on the question of how molecular mechanisms control the ciliary length during ciliary assembly, maintenance and disassembly. Recently, Avasthi et al. and Izawa et al. published comprehensive reviews about proteins and pathways that influence the ciliary length. They describe a crosstalk between the formation of the PC and cell division [20,32]. Furthermore, a molecular link (CDK5-FBW7-NDE1 pathway) between ciliary length and cell cycle has been observed [34]. Thereby, the ciliary length is not only a passive state that depends on external influences. The length itself was described as a “checkpoint” for cell cycle re-entry [32,39].

### 2.3. Intraflagellar Transport (IFT)

During ciliogenesis the PC is formed by the elongation of the axoneme and protrusion of the cell membrane. Axonemal building blocks and ciliary membrane proteins need to be arranged in a specific architecture to guarantee the proper function of the extended PC. Consequently, the synthesis needs to be well regulated. The biosynthesis of proteins occurs at the endoplasmic reticulum and trafficked to the PC via the Golgi apparatus. When protein arrives at the BB of the PC it is essential that protein transport mechanism guarantee the correct transport and arrangement of ciliary proteins. Visualizations of moving proteins along the axoneme were first performed with video enhanced differential interference contrast (DIC) microscopy by Kozminski et al., who detected membrane and axoneme associated motilities of granule-like particles in the flagellum of the green alga *Chlamydomonas reinhardtii*, a well-established model for ciliary research [41]. This transport mechanism was called ‘Intraflagellar Transport’ (IFT) [41]. It describes a bidirectional motility system, which works continuously with connection to the axoneme [42,43]. IFT is the main mechanism that is responsible for assembling, maintaining and disassembling of PC during the cell cycle. Pedersen et al. published a model for IFT in which the whole process is described to be a cycle of six steps. The first step includes the gathering of IFT motor and complex proteins around the BB. Next, Kinesin II is activated and transports proteins to the ciliary tip. In the third step the motor proteins reach the tip of the PC and the transported proteins are dissociated and released. Kinesin II is inactivated and Dynein II is activated and binds with IFT protein complex A. The retrograde movement of Dynein II to the ciliary base represents the fifth step of the model. The cycle closes with the recycling of the IFT components in the cytoplasm [43].

Recent studies on model organisms revealed several processes and proteins that relate to IFT. IFT associated proteins can be grouped as IFT motor proteins, IFT complex proteins and accessory proteins [27]. IFT motor proteins guarantee the continuous movement along microtubules to ensure the protein transport in the anterograde as well as in the retrograde direction. The anterograde transport is managed by heterotrimeric Kinesin II motor proteins (KIF3A and KIF3B in mammals) and homodimeric Kinesin II proteins (KIF17 in mammals) [27,42,44,45]. The retrograde transport is performed by cytoplasmic Dynein II proteins (DYNC2 in vertebrates) [27,42,43,44,45]. Together with IFT complex proteins, IFT motor proteins are able to form “IFT trains” - multimeric protein complexes with the capacity to transport a variety of cargo molecules [45]. In such formations IFT motor proteins are attached to chains of IFT complex proteins that carry the cargo molecules. IFT complex proteins are divided in two subgroups—IFT complex A, which consists of six subunits (IFT-43, -121, -122, -139, -140, -144) and IFT complex B, which consists of 16 subunits (IFT-20, -22, -25, -27, -38, -46, -52, -54, -56, -57, -70, -74, -80, -81, -88, -172) [27,42,43,45]. The importance of each complex protein is reviewed elsewhere but to give an overview, it varies from flagellar/ciliary formation to the regulation of osteogenesis, signaling and mechanosensing [29,42]. All these functions are regulated, coordinated and controlled by several IFT accessory proteins. To date, over 60 accessory proteins have been identified [44]. This protein family includes Pericentrin, Centrin 2, PCM-1 and Alms1, which are required for ciliary assembly in the early steps of ciliogenesis [43]. The BBSome, a complex of eight proteins, assembles the IFT complex proteins at the base of the PC [44,46].

With respect to its importance, IFT is not only guaranteeing a dynamic ciliary assembly and disassembly during ciliogenesis and cell cycle. IFT proteins are also involved in exocytosis, trafficking and selective cargo loading at the TZ serving as a ciliary gate [27,44,45,47]. IFT is also involved in several signaling pathways concerning skeletal development as well as cartilage and bone homeostasis [29,44,46,48,49,50].

### 2.4. Morphological Features of the Growth Plate

The skeletal development of long bones is a result of endochondral ossification, a process in which growing cartilage is replaced by bone. This involves the growth plate (GP) that is responsible for 80% of the long bone growth and represents a key structure during skeletal development [51]. Morphologically, the GP is a highly organized structure with four zones—the reserve proliferative hypertrophic and invasive zones [52,53,54]. The reserve zone is characterized by the condensation of mesenchymal stem cells (MSC), which differentiate to chondrocytes under the influence of growth factors [50,52,55,56]. In the proliferative zone chondrocytes organize themselves through cell divisions and rotation [52,53,56,57]. By this they form columns of 6-8 flat and stacked cells, unidirectional along the longitudinal axis of bone formation [50,52,53,58]. In this stage the chondrocytes secrete type II collagen [50,52,56,58]. Afterwards, the cells pass through a pre-hypertrophic phase [58,59]. The following hypertrophic zone is mostly defined through cell differentiation [52]. Here, the cells secrete ECM proteins, like type X collagen and Ca^2+^ and thereby are actively involved in the mineralization of the matrix [52,55,56,58]. In addition, they attract osteoblasts and blood vessels to the scene [50,52,55,56]. Finally, in the invasive zone, osteoblasts manage the remodeling and ossification of the tissue, while chondrocytes undergo apoptosis [50,52,55].

### 2.5. Links between Primary Cilia and Tissue Organization During Skeletal Development

**Figure 4:**
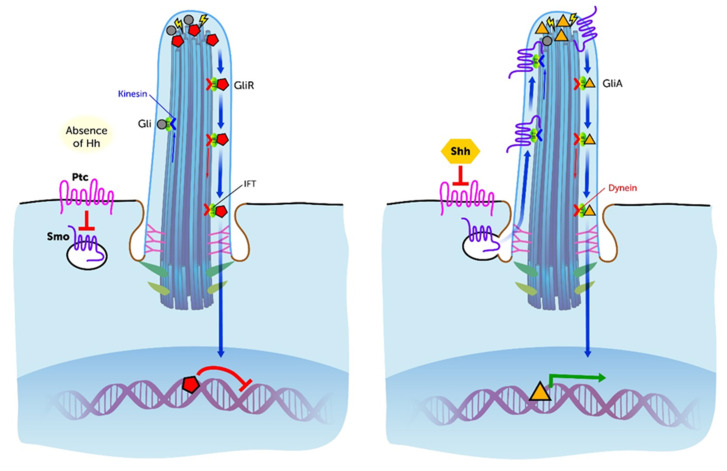
The physiological structure and functioning of the Primary Cilium are prerequisites for proper Shh pathway which is responsible for an adequate skeletal development, tissue patterning and thus an ordered cartilage and bone growth. Particularly, the Intraflagellar Transport (IFT) associated proteins are of high importance in this respect. Without Shh signal gli proteins (grey circles) are activated (yellow flashes) to their repressor form (red pentagons) by the inhibition of Smo (purple wavy line) via the Ptc receptor (pink wavy line). In the presence of Shh (yellow hexagon) Ptc is blocked and Smo is released to the cilium, where gli proteins are activated to their transcriptional activator forms (orange triangles). The anterograde and retrograde transport function along the Axoneme via IFT motor proteins (Kinesin and Dynein) enables the translocation of the Gli transcription factors to the nucleus. Thus, the transcription of important genes during skeletal development is regulated. Disorders within this complex cascade lead to skeletal abnormalities. Image modified from Singla & Reiter (2006) [5].

Several ciliary and IFT associated pathways coordinate the osteo- and chondrogenesis during embryological and post-natal development of the GP. Especially the importance of Hh signaling during embryonic skeletal development is well established (Figure 4). The mammalian Hh proteins are Sonic Hedgehog (Shh), Indian Hedgehog (Ihh) and Desert Hedgehog (Dhh) [53]. At the ciliary site Hh binds to its receptors Ptc1 or Ptc2 (Patched 1, 2). This releases Smo (Smoothened) that is translocated in the anterograde direction to the ciliary tip. This activates Gli transcription factors, which are transported retrogradely along the axoneme to the cell’s body. Here they initiate gene expression in the nucleus of the cell. This signal cascade is essential for proper bone formation and tissue patterning [55]. In vivo studies murine models lacking ciliary proteins such as IFT88, IFT80, IFT20 and Kif3A (ciliary motor protein) have demonstrated a direct and indirect role for PC and IFT in the organization and development of chondrocytes in the GP. Haycraft et al. clearly demonstrated that Shh is crucially connected with the limb development since the disruption of the Shh pathway by IFT88 deficient mice, caused a loss of tissue patterning and resulted in polydactyly [55]. Ihh is necessary for the proliferation of chondrocytes and differentiation of osteoblasts through the Hh pathway [51,55,56]. The interruption of Ihh signaling leads to mis-orientation and failed rotation of chondrocytes in the pre-hypertrophic zone resulting in a shortening of the long bones [55]. It has also been demonstrated that PC regulate the planar cell polarity (PCP) of chondrocytes, which is a significant process within the columnar formation in the proliferative zone of the GP [53]. Here, β-1 Integrins, Hh and the non-canonical Wnt signaling are involved in the specific emergence of PCP and therefore for cell orientation and cell migration [56,57,60]. However, the β-Catenin associated canonical pathway is also important for the differentiation of osteoblasts [56]. These findings are in line with the analyses of McGlashan et al. who investigated the role of PC during skeletal development on ORPK mice. These mice revealed mutations in the *POLARIS* gene resulting in a loss of IFT88. The animals showed significantly smaller GPs with less cellular columns, less flattened cells and reduced type X collagen in the hypertrophic zone [55]. It was also pointed out that pre-hypertrophic chondrocytes secrete Ihh, which leads to an induction of Wnt signaling and induces chondrocyte differentiation, which is negatively regulated by the parathyroid hormone-related peptide (PTHrP) [54,55,58,59,61]. The mechanisms of chondrocyte differentiation in the GP are controlled by several feedback mechanisms which supports the theories of Olsen et al. who described Ihh and PTHrP as regulative components for the relative proportions of the chondrocyte proliferation and hypertrophy in the GP [51]. Song et al. showed with experimental studies in Kif3A deleted mice, if PC were depleted, chondrocytes undergo a reduced proliferation and accelerated differentiation, which finally results in dwarfism. Furthermore, they found alterations in the rotation of chondrocytes with interrupted columnar formations in the proliferative zone of the GP [50]. Koyama et al. demonstrated that the conditional Kif3A ablation in mice leads to disturbed chondrocyte proliferation and hypertrophy with subsequent GP disorganization and cranial base dysmorphogenesis [62]. In addition, these experiments affected the collagenase MMP-13 (=matrix metalloproteinase 13 also known as collagenase-3) levels [62]. MMP-13 is a key protein for the cartilage matrix catabolism [63]. It is responsible for chondrocyte proliferation, cellular migration, cell apoptosis, degradation of collagen II, alterations of the ECM and the cartilage to bone transformation [64]. Thus, MMP-13 play critical roles during GP formation and skeletal development. More recent studies demonstrated fibroblast growth factor (FGF) regulates the post-natal bone growth by regulating the autophagy of chondrocytes [65]. Autophagy, in turn, has the capacity to suppress ciliogenesis [66,67]. Noda et al. found an interconnection between IFT20 and the craniofacial development. In their experiments IFT20 deletion in mice lead to reduced PDGFα expression, which is important for osteoblast differentiation [68]. Furthermore, it has been shown that mesenchymal stem cells (MSC) lacking cilia express decreased numbers of transcription factors and thereby limit the initiation to differentiate to its phenotype [46]. This observation underlines the active role of PC in cell differentiation. It has been shown that PC and IFT proteins take over important tasks during skeletal development, especially for MSC, chondrocytes and osteoblasts. Taken together, these findings indicate that PC are directly or indirectly connected to a proper proliferation, differentiation, rotation, orientation and migration of the mesenchymal cells via several signaling molecules and pathways [55]. Thus, the structural and functional integrity of PC guarantees the correct tissue patterning and limb development during embryological stages and care for correct bone enlargement and maintenance in post-natal growth phases.

## 3. Pathophysiological Aspects of Primary Cilia

### 3.1. Ciliopathies

One of the first relationships between a PC defect and the occurrence of a disease was identified in Polycystic Kidney Disease (PKD). Mutations in *PKD1*, *PKD2* (autosomal dominant PKD) or *PKHD1* (autosomal recessive PKD) lead to a loss of regulatory function and defective ciliary signaling [1,5,11,69,70,71]. The affected genes encode for proteins are localized at the PC of the renal epithelial cells—Polycystin 1, Polycystin 2 and Fibrocystin respectively [70]. The underlying pathophysiological mechanisms are complex and are the subject of ongoing research [70]. As a consequence of PCP disruption a disorganization of kidney epithelial cell growth occurs and ends in an excessive proliferation of the kidney tubules to multiple cysts [1,5,70,71]. Subsequent to the linking of PKD to the PC several other PC associated genes have been linked to disease [12,13]. These inherited pathologies are termed ciliopathies [10,11,12,13]. Ciliopathies refer to a large group of rarely occurring diseases and syndromes with different genotypes but often an overlap in phenotype [11]. Skeletal anomalies, such as polydactyly, craniofacial abnormalities, chest deformities or changes in bone length are a feature of many ciliopathies [50]. Congenital skeletal disorders are described for Meckel-Gruber [72], Bardet-Biedl [73], Oral-Facial-Digital Syndrome Type 1, Mc Kusick-Kaufmann and Short-Rib Polydactyly (SRP) Syndromes [11]. The SRP-Syndromes themselves represent a subgroup of ciliopathies. Their clinical occurrences are characterized by different severities of skeletal anomalies and extra-skeletal phenotypes. Mc Inerney et al. classified the SRP-Syndromes in two groups. On the one hand, there are syndromes with a milder phenotype, like Jeune-Syndrome, Ellis van Creveld Syndrome, Sensenbrenner Syndrome and Asphyxing Thoracic Dystrophy [74]. On the other hand, there exist SRP-Syndromes with severe phenotypes may lead to congenital lethal disorders [74]. The latter forms were subclassified in five types and include Saldino-Noonan Syndrome (Type 1), Majewski Syndrome (Type 2), Verma-Naumoff Syndrome (Type 3), Beemer-Langer Syndrome (Type 4) and a no named Type 5 [75]. SRP-Syndromes can be caused by heterozygous mutations in several genes, including *WDR60* and *WDR35* [11,74,75]. Defects of *WDR60* express themselves in a disorganized ciliogenesis and faulty Gli2 transcription factors [74]. Gli transcription factors are also connected with Hh signaling and therefore with tissue patterning and cell proliferation [5]. So, *WDR60* is a further example for the involvement of PC in skeletal development and cartilage tissue organization. The SRP-Syndromes also include extra-skeletal symptoms, for example cardiac, liver and brain anomalies. These facts underline the systemic character of ciliopathies and the importance of proper functioning of PC in various tissues.

### 3.2. Ciliary Orientation in Cartilage—Expression of Pathophysiological Changes?

Mature articular cartilage is a highly organized, anisotropic tissue with a characteristic division in three zones [76]. In this tissue, PC appear one per cell, emerge perpendicular from the center of the cell’s major or minor axis and point in a special or unequal direction, depending on the zone and area [21,77,78]. The deep zone of articular cartilage, which adjoins the subchondral bone, is determined through large chondrocytes, which contribute to the ECM by matrix secretion [79]. Here, the PC point in multiple directions and thus, do not follow an ordered pattern [76,80]. In the transition zone the chondrocytes are arranged in columnar stacks longitudinal to the articular surface [76]. Typical ECM proteins, like aggrecan and collagen II are secreted in this zone. PC in the transition zone either point to the subchondral bone or towards the articular surface [76]. The superficial zone is morphologically defined by small elongated cells [79]. The long axis through these chondrocytes is oriented parallel to the articular surface [78]. PC in the load bearing areas of the superficial zone consistently point away from the articular surface to the subchondral bone [53,76]. In less load bearing areas, the orientation of PC is less organized. With respect to the elucidation of tissue organization and ciliary alignment in the GP, it seems plausible the architecture of mature connective tissues also relates to the ciliary orientation. In 3-D-multiphoton microscopic analysis of the incidence and orientation of PC, Donelly et al. could demonstrate 64% of tenocytes within tendons express a PC, which is oriented in the same direction as the collagen fibers. They hypothesized there is a coincidence between the orientation of PC, of the ECM and the loading direction of the tendon [81]. In bone tissue, Uzbekov et al. discovered that PC of osteocytes oriented themselves perpendicular to the long axis of the considered bone [82]. In articular cartilage the direction of the PC also matches with the orientation and organization of the ECM [81]. Various studies about pathological transformed cartilage showed changes in ciliary orientation, length and incidence. Rich et al. found a more diffuse orientation of PC in calcified cartilage compared to healthy tissue [18]. In Osteoarthritis, PC of cells in the upper zones of articular cartilage increase in numbers and lengths with the severity of the disease [83]. The etiology of this effect has not been clarified. In the transition zone, the chondrons transform to clusters and the PC align themselves to the center of those clusters [83]. Studies on Col2Cre;Ift88 deleted transgenic mice, who lost their PC, showed relevant morphological changes with an increase of osteoarthritis (OA) markers such as thickening, reduced stiffness and abnormal joint formation [29,84,85].

**Figure 5:**
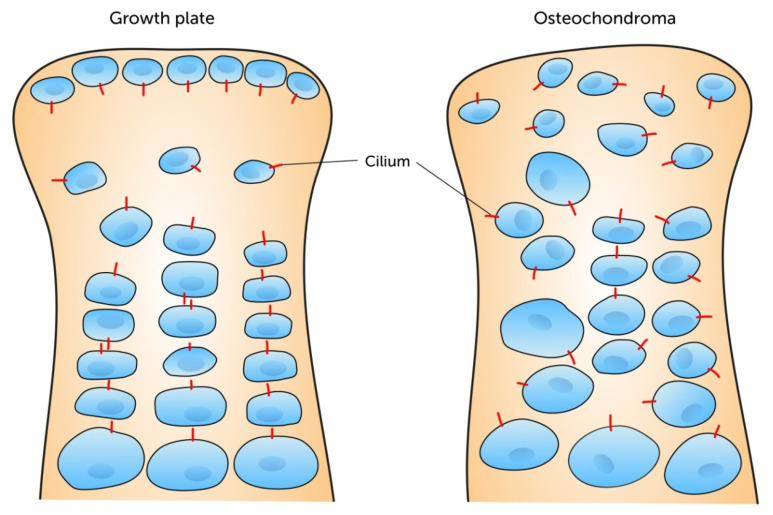
The ordered tissue morphology in healthy growth plates is accompanied by a certain pole-related ciliary orientation (chondrocytes given in blue, cilia given in red). In contrast, the disorganization of chondrocytes in osteochondromas is associated with a disordered ciliary alignment. Image modified from McGlashan et al. (2007) [58].

Taken together, these findings indicate a possible involvement of PC in pathophysiological processes such as the development of OA [14]. Nevertheless, changes of the ciliary orientation not only seem to play a role in OA but also in neoplastic changes of the connective tissues. De Andrea et al. published several studies regarding ciliary misorientations in osteochondroma. Using confocal and electron microscopy they detected a random orientation and shorter PC, compared to healthy articular cartilage. They hypothesized the ciliary orientation reflects a cellular polarity is cancelled in the occurrence of neoplasia [61]. More recently they showed in mouse models with chondrosarcoma and enchondroma PC are misoriented and this abrogation is interconnected with a poor organization of chondrocytes (Figure 5) [86]. Furthermore, in chondrosarcoma cells Ho et al. showed decreased numbers of PC (12.44%), compared to normal tissue (67.7%) [87].

Taken together, these findings indicate a possible structural connection between the orientation of chondrocyte PC and the special architecture of articular cartilage. The specific orientation of PC suggests that PC are involved in the positioning and rotation of chondrocytes in the three-dimensional space. The defined arrangement of PC of the main load bearing areas of the superficial zone and the transition zone could be a morphological adaptation to the mechanical stress on the tissue. Especially in the load-bearing areas of the superficial zone the PC positions itself below the cell body, possibly in order to use it as a protective shield against mechanical overload. Thus, the PC could protect itself from damage or superfluous mechanosensitive signaling to maintain its proper functions. In addition, it is discussed that the ciliary positioning of chondrocytes could be an effort to protect special intracellular structures from mechanical forces [18], which supports the theory of a protection function of the PC hypothesized by McGlashan et al. [88].

### 3.3. Morphological Adaptations of PC

Mechanical loading on cartilage transfers directional forces through the tissue. To protect the articular cartilage against damage the forces need to be buffered by distribution in the high specialized cell-matrix composition. The compression of the ECM leads to a passive ciliary bending, which is associated with cell signaling [14,21]. Electron microscopic imaging showed bending patterns and contact points of PC to structures of the ECM [14,21]. This observation indicated that chondrocyte PC could have a mechanotransducive effect as it is also known for PC on renal epithelial cells. Even in bone, confocal microscopic studies discovered bending patterns of osteoblast PC, which gives clues about mechanosensitive functions of PC in this tissue [53]. ‘Mechanotransduction’ is a cellular function, which Papachroni et al. defined as a triad of mechanosensing, signal transduction and cellular response [89]. It has been shown that mechanical strain on chondrocyte PC induces an up-regulation of components of the ECM [21,90]. In this context, it was described that chondrocytes in agarose constructs react on mechanical loading via intracellular Ca^2+^ signaling [91]. TRPV4 channels, which are located at the ciliary membrane, are involved in intracellular Ca^2+^ increase and subsequent metabolic responses, like ADAMTS5 gene expression which has been linked to the of osteoarthritis [92,93,94]. McGlashan et al. showed that the mechanical loading has an influence on the ciliary length and incidence, proposing this is an adapting mechanism of PC. They showed the PC adapt by decreasing in length and incidence when there is an overbearing or prolonged signals [88]. Wilsman et al. determined the occurrences of PC in equine and murine chondrocytes with electron microscopic serial sections. They discovered rates of 96% in equine and 100% in murine chondrocytes [95]. McGlashan et al. analyzed bovine articular chondrocytes and found a 46% incidence of PC in healthy cartilage [83]. Ho et al. examined rates of 67% in human articular cartilage [87]. These differences could of course be caused by the different investigated species. Nevertheless, McGlashan et al. showed in a three-dimensional agarose model length and incidence of PC are significantly reduced in compressive strain exposed chondrocytes which gives a link to the pathophysiology of osteoarthritis [88]. Concerning the ciliary length, Saggese et al. demonstrated that the calculating of ciliary lengths in 2-dimensional models underestimates the true ciliary lengths. They present a method to measure it in a 3-dimensional way. With their experiments they detected values between 2 and 50 µm in wild type murine chondrocytes [96]. Ex vivo experiments with murine femoral chondrocytes by Rich and colleagues revealed an average length of PC from 0.81 µm (± 0.24 µm). However, their experiments also demonstrated that the ciliary length may change within minutes. According to the authors the ciliary length changes in response to osmotic changes in the ECM [18]. The observation that IL1β increases cilia length in bovine articular chondrocytes also indicates a link between inflammatory processes and PC elongation [97]. Spasic et al. suggested a ciliary length and stiffness relationship that could likely dictates the mechanosensitivity of PC [90]. Furthermore, the ciliary length is dependent on the mechanisms of IFT and therefore it is associated with several signaling pathways [27]. Moreover, the ciliary length relates to the molecular mechanisms during cell cycle and ciliogenesis and their complex molecular coordination.

The comparison of PC in normal articular cartilage and pathological transformed tissue revealed PC and their functions are required for a regular tissue organization and proper tissue maintenance. Despite these investigations, it remains unclear whether ciliary defects actively cause tissue transformations or if the ciliary orientation and length changes are passive mechanisms to adapt to extracellular alterations. To detect further physiological and pathophysiological roles of PC in articular cartilage future studies need to be performed with focus on the pathophysiologic role and molecular mechanisms of PC in OA or cartilage neoplasia.

### 3.4. Molecular Links between PC and Osteoarthritis

**Figure 6:**
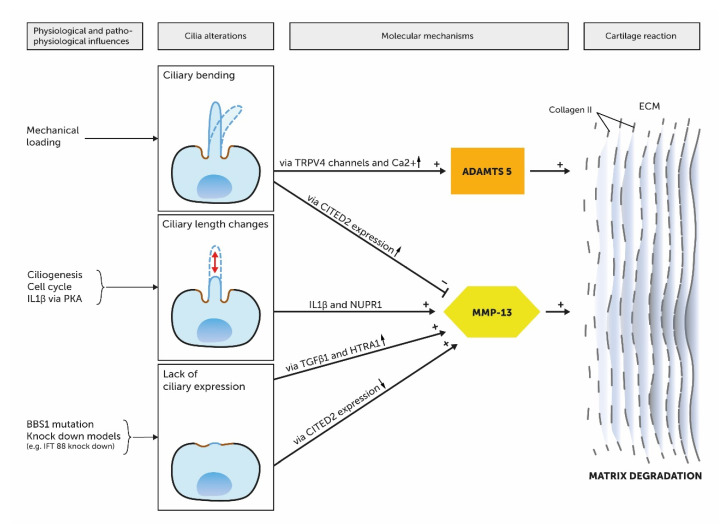
Short summary of the ciliary involvement in molecular mechanisms in the context of OA. Cilia alterations are influenced by physiological and pathophysiological processes and affect molecular mechanisms of OA. Ciliary bending leads to increased calcium levels via TRPV4 channels and subsequently to an activation of the ADAMTS5 gene expression. Furthermore, cyclic mechanical loading can suppress MMP-13 via upregulation of CITED2 expression. In addition, ciliary length changes were reported in the context of OA. IL1β treatment leads to an elongation of the cilium via Proteinkinase A (PKA). IL1β in interaction with NUPR1 results in increased MMP-13 expression. Experimental knock down of ciliary genes lead to a lack of ciliary expression and subsequently to an activation of MMP-13 expression for example via increased HTRA1 levels (BBS1 mutations) or via downregulation of CITED2 expression (IFT88 knock down).

The pathophysiology of OA is understood as an imbalance between anabolic and catabolic factors, which leads to cartilage degradation. This imbalance is induced by aging, inflammation and biomechanical factors in turn relate to PC through mechanotransduction and other molecular mechanisms [14,98]. In this context, increased matrix metalloproteinase 13 (MMP-13) levels play a critical role in the pathophysiology of OA [99]. MMP-13 cleaves collagen II and leads to a degradation of the ECM [100]. Several pathways are known to activate MMP-13 gene expression (Figure 6). On the one hand, inflammatory signals in form of IL-1β in interaction with stress inducible nuclear protein 1 (NUPR1) leads to an increased expression of MMP-13 [100]. In this context, Wann et al. demonstrated that the ciliary length was increased through a treatment with IL-1β in human chondrocytes via Proteinkinase A (PKA) and suggest a link between inflammation and PC in OA [101]. On the other hand, stress on chondrocytes leads to an increase of MMP-13 via transforming growth factor β1 (TGF-β1) mediated binding of high temperature receptor A1 (HTRA1) to the discoidin domain-containing receptor 2 (DDR-2) [100]. Sheffield et al. suggest PC to be involved in the TGF-β1/HTRA1/DDR-2 axis. Experiments with BBS1 mutant mice showed subsequent defective PC that had increased HTRA1 and MMP-13 levels [100]. Furthermore, the transcription factor Hes1 is involved in the upregulation of MMP-13. This is mediated via the Notch signaling pathway which modulates Shh signaling that in turn is transduced along the PC [100]. In this context, it was a matter of debate, if there is a potential association between the expression of pro-catabolic MMP-13 and ADAMTS-5 and the ciliary Ihh pathway. Thompson et al. discovered that the expression of MMP-13 and ADAMTS-5 is neither dependent of Ihh nor in coexistence with IL-1β [97]. However, Tao et al. showed disturbed ciliary function via knock out of the ciliary motor protein Kif3a in mice leads to altered MMP-13 levels [63]. He et al. described another PC-associated signaling pathway that leads to increased MMP-13 and MMP-1 levels. They reported that cyclic stress on human chondrocytes leads to a Cbp/p300 Interactin Transactivator with ED-rich tail 2 (CITED2) expression that suppresses MMP-1 and -13 [102]. IFT88 knock-down decreased CITED2 expression but upregulated MMP-1 and MMP-13 expression [102]. Furthermore, PC associated factors TGF-β3 and PIEZO ion channels play anabolic (TGF-β3) and catabolic (PIEZO ion channels) roles in the pathophysiology of OA [19,98,103]. Further studies focused on the biomechanical aspects of PC in OA. O’Conor et al. demonstrated in their experiments that TRPV4-gene knockout mice showed reduced severity of aging associated OA [98]. In summary, these investigations make clear that the pathophysiological mechanisms of OA relate to PC.

## 4. Conclusions

Numerous studies have shown a variety of functional and structural relationships between PC and physiological as well as pathological aspects of developing and mature articular cartilage. The PC pick up signals from the cellular environment and serve as a structural interface for signal cascades, enabling adequate cell and ECM growth. PC are essential for an adequate tissue development, organization and homeostasis. The current literature provides indications of possible morphological relationships between PC and the pathophysiology of osteoarthritis. Nevertheless, the pathophysiological role of PC in osteoarthritis is still being discussed and should be addressed more specifically in future research.

## Abbreviations

BB	Basal body
CCV	Clathrin coated vesicles
CM	Ciliary membrane
CP	Ciliary pocket
Dhh	Desert hedgehog
ECM	Extracellular matrix
GP	Growth plate
GPCRs	G-Protein coupled receptors
Hh	Hedgehog
Ihh	Indian hedgehog
ITF	Intraflagellar transport
MSC	Mesenchymal stem cells
OA	Osteoarthritis
PC	Primary cilium / Primary cilia
PKD	Polycystic kidney disease
Shh	Sonic hedgehog
TE	Tissue engineering
TF	Transition fibers
TGF-β	Tissue growth factor β
TZ	Transition zone
